# snp-search: simple processing, manipulation and searching of SNPs from high-throughput sequencing

**DOI:** 10.1186/1471-2105-14-326

**Published:** 2013-11-19

**Authors:** Ali Al-Shahib, Anthony Underwood

**Affiliations:** 1 Applied Bioinformatics and Laboratory Informatics Unit, Microbiology Services, Public Health England, 61 Colindale Avenue, London NW9 5EQ, UK

**Keywords:** Single Nucleotide Polymorphisms (SNP), Variant Call Format (VCF), SQL database, High-throughput Sequencing, Next Generation Sequencing (NGS), Ruby, Phylogeny

## Abstract

**Background:**

A typical bacterial pathogen genome mapping project can identify thousands of single nucleotide polymorphisms (SNP). Interpreting SNP data is complex and it is difficult to conceptualise the data contained within the large flat files that are the typical output from most SNP calling algorithms. One solution to this problem is to construct a database that can be queried using simple commands so that SNP interrogation and output is both easy and comprehensible.

**Results:**

Here we present snp-search, a tool that manages SNP data and allows for manipulation and searching of SNP data. After creation of a SNP database from a VCF file, snp-search can be used to convert the selected SNP data into FASTA sequences, construct phylogenies, look for unique SNPs, and output contextual information about each SNP. The FASTA output from snp-search is particularly useful for the generation of robust phylogenetic trees that are based on SNP differences across the conserved positions in whole genomes. Queries can be designed to answer critical genomic questions such as the association of SNPs with particular phenotypes.

**Conclusions:**

snp-search is a tool that manages SNP data and outputs useful information which can be used to test important biological hypotheses.

## Background

Next Generation Sequencing (NGS) technologies have facilitated large scale resequencing of genomes for the purpose of Single Nucleotide polymorphism (SNP) discovery. The thousands of discovered SNPs during such projects can cause some difficulty for biological interpretation and computational analyses.

To address this challenge, various databases and tools have been created that annotate SNPs with information derived from a reference genome and store that information within a database [1-7].

While these tools have excelled at the management of the SNP data, less attention has been directed towards simple processing and searching of the stored data. For example, the tool developed by Wang *et al* for SNP management includes limited search functions and does not allow for advanced user database querying. Some databases like that described by Dereeper *et al* have implemented a web tool for SNP discovery and analysis. However, it does not accept Variant Call Format (VCF) files as input and is only applicable to diploid species. Another tool is SNPpy by Mitha *et al* which provides a database management system that allows for SQL querying and data manipulation. The SNPpy system is dependent on a relational database (PostgreSQL) that requires considerable expertise to administer and does not accept VCF. VCF files were an initiative from the 1000 Genomes Project [8] and are an output produced by many SNP calling programs such as dbSNPs [9], Samtools mpileup [10] and GATK [11].

There is a need therefore for a tool that extracts SNPs from VCF files, stores them into a simple database and provides multiple output options for analysis. To meet such a need, we have developed snp-search, an easy to use tool for management of SNPs generated from haploid next generation sequencing data. Given a VCF file, snp-search stores the SNPs generated by the variant calling algorithm into a sqlite database. Sqlite is a relational database contained in a single file and does not require complex management software. snp-search can then be used to extract useful information from the database. For example, by running snp-search using the command line syntax, the user can extract unique SNPs for a specified set of strains; generate a SNP phylogeny and provide detailed information about each individual SNP such as if it is a synonymous or non-synonymous SNP, gene information, etc (see Table 1). The snp-search database schema has been purposely designed to be simple so that it is easy to interpret findings from large and complex biological queries. Therefore a simple SQL join is able to extract data that will provide the power to answer questions about variation within the core genome quickly and easily. snp-search also has the ability to filter SNPs depending on the function of genes within which the SNPs are found. Furthermore there is no file size limit and no dependence on any external tool. The sole requirements are a Unix environment, SQLite, Ruby 1.8.7 or greater and optionally FastTree 2 [12] for phylogenetic tree construction. As far as we are aware, there are no available tools with similar features as snp-search in the literature.

**Table 1:** ST315 clade unique SNP information - typical snp-search output for –u option

**Pos**	**Ref base**	**SNP**	**syn/non-syn**	**Gene annotation**	**Pseudo gene?**	**AA orig**	**AA change**	**CH?**	**CP?**	**CS?**
16387	A	G	syn	putative amino acid permease	No					
512693	A	G	non-syn	putative dipeptidase	No	T	A	No	Yes	No
924493	T	C	non-syn	putative sugar ABC transporter (ATP-binding protein)	No	S	P	No	Yes	No
1108998	G	T	non-syn	putative N-acetylglucosamine-6-phosphate isomerase	No	A	S	Yes	Yes	No
1186147	C	T	syn	putative amino acid ABC transporter (ATP-binding protein)	No					
1573272	T	C	non-syn	putative sucrose operon repressor	No	L	S	Yes	Yes	Yes
1817279	C	T								
1868362	T	C								

Last two empty cells indicate the SNP has occurred in a non-CDS region therefore having no further information. Syn: synonymous, Pos: position of SNP in reference genome, AA: Amino acid, orig: original, CH: change in hydrophobicity, CP: change in polarity, CS: change in size.

## Implementation

snp-search is written in Ruby, a popular scripting language, and uses the Ruby ActiveRecord library that maps database tables to Ruby objects. It will run on most modern Unix-based architectures and can be installed with the command *gem install snp-search*.

snp-search has two fundamental features:

**Figure 1:**
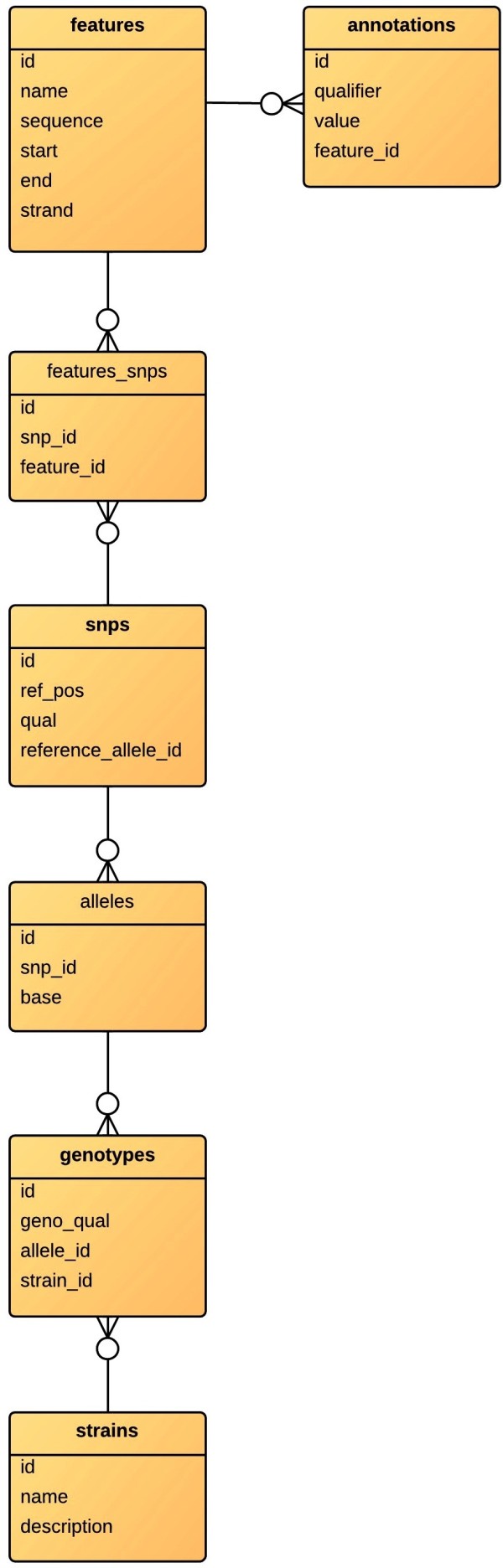
snp-search database schema.

**Figure 2:**
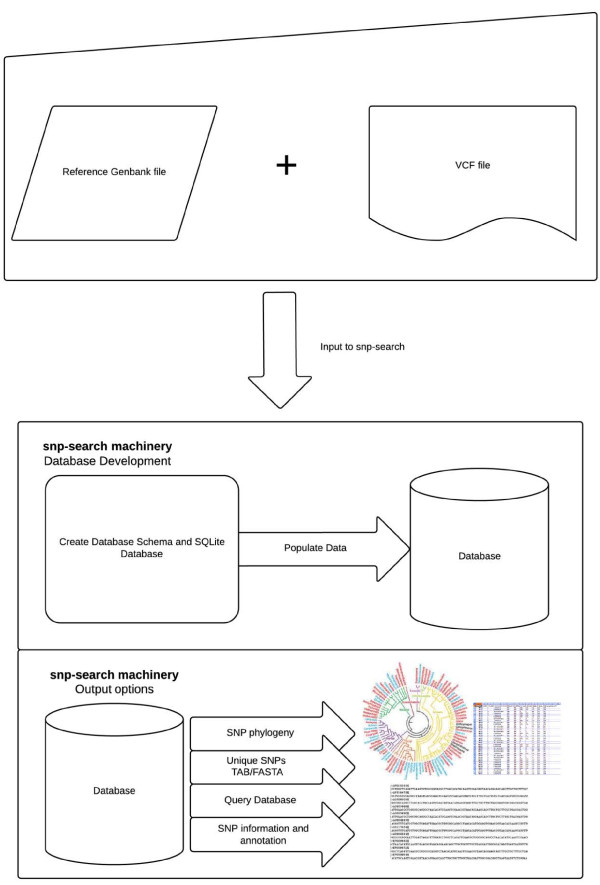
**Snp-search machinery.** Given a genbank and a VCF file, snp-search first creates the SQLite database and populates the data into the database. The user will then have the choice of various output options. Options include producing a SNP concatenated FASTA file, generating a newick-tree format file for phylogenetic analysis and a list of SNPs (depending on query) with information for every SNP (such as if the SNP is synonymous or non-synonymous).

1. Creation of a local SQLite database and schema

To create the database, data is sourced from two input files: reference genome (in genbank or EMBL format) and a VCF file. snp-search requires the reference genome used in the mapping process to import the features (genes, etc) and annotations into the database and requires the VCF file to populate the database tables with the SNP information.

The database schema created by snp-search is designed to facilitate construction of simple queries that will address complex biological questions. Annotations from the genbank file are populated in the *annotations* table which is related to the *features* table which contains information on the type of the feature and its nucleotide sequence. SNP positions from the VCF file are imported to the *snps* table which is related to the *features* table by the *features_snps* table. A SNP that has occurred in each resequenced genome is recorded as an allele in the *alleles* table. The strain name and description (*strains t*able) is related to *alleles* via the *genotype* table which contains the *allele_id* and *strain_id.* Thus queries that relate *strains* to *snps* or *annotations* may be easily composed. These relationships are schematised in Figure 1.

2. Output requested objects from database

Once the database has been populated, snp-search provides several filtering and output options (Figure 2).

### Output formats

#### Concatenated FASTA file

SNPs for each sample are collected from the database and concatenated and converted to FASTA format.

#### Tab-delimited tabular file

Information for individual SNPs are provided in tab-delimited tabular format.

#### Newick-file format

snp-search uses FastTree 2 [12] to generate a Newick file for SNP phylogeny.

### Filtering options

#### Filter SNPs according to the SNP Quality

A Phred-scaled score that the SNP exists at the given site (default 90).

#### Filter SNPs according to the Genotype Quality

A Phred-scaled score that the genotype is true (also known as Genotype quality score, default 30).

#### Filter SNPs according to the AD ratio

The ratio of the unfiltered count of all reads that carried that specific allele compared to other REF and ALT alleles in that site (default 0.9).

#### Ignore SNPs from feature

Ignoring particular SNP calls in features, such as phages, transposases, and insertion sequences to remove horizontally transferred DNA as a result of a recombination event.

#### Ignore SNPs in specified range

SNPs in a defined specified range will be ignored.

#### Ignore samples

Certain samples can be excluded from the SNP output.

#### Ignore non-informative SNPs

A SNP that is found in all samples is not included in the output.

### Analysis

#### Unique SNPs for a set of strains

Given a set of defined strains as input, snp-search queries the database for unique SNPs shared by the defined strains and outputs the results in concatenated FASTA or tabular format.

#### SNP phylogeny

Concatenated FASTA files are used to generate robust phylogenetic trees that are based on SNP differences across whole core genomes.

#### SNP annotation

snp-search processes the data in the database and outputs information on each SNP. The following are the description of this output:

– **Synonymous or non-synonymous SNP.** This is calculated by translating the coding sequence and report if the SNP has whether caused an amino acid change or not.

– **Function of coding region at the SNP position.** This is extracted from the annotated genbank file.

– **Possible pseudogene.** If a stop codon is detected in the coding region (other than the end of the sequence).

– **Original amino acid.** Provide the amino acid for the SNP region in the non-mutated sequence.

– **Has amino acid has changed due to the mutation?** An answer of 'yes’ will be given if there are at least one amino acid changes between the original non-mutated sequence and the mutated sequence.

– **Is there a change in hydrophobicity of the amino acid?** The answer to this question is based on the pre-defined set of hydrophobic and non-hydrophobic amino acids taken from Livingstone et al.*,*[13]: hydrophobic as [I,L,V,C,A,G,M,F,Y,W,H,T] and the non-hydrophobic amino acids as [K,E,Q,D,N,S,P,B].

– **Is there a change in the polarity of the amino acid?** The answer to this question is based on the pre-defined set of polarised and non-polarised amino acids taken from Hausman et al., [14]: polar as [R,N,D,E,Q,H,K,S,T,Y] and non-polar as [A,C,G,I,L,M,F,P,W,V].

– **Is there a change in size of the amino acid?** The answer to this question is based on the pre-defined set of small or non-small set of amino acids taken from Livingstone et al.*,*[13]: small as [V,C,A,G,D,N,S,T,P] and non-small as [I,L,M,F,Y,W,H,K,R,E,Q].

#### Database query

SQLite database can be interrogated by user defined queries (requires some knowledge of SQL). To view, administer and query the database, one may download a SQL GUI tool or simply use the Unix version of sqlite3.

## Results and discussions

### Application

The snp-search tool was used to generate a SNP database of 200 Group A *Streptococcus pyogenes* strains (GAS) sequenced using 50 bp reads on the Illumina HiSeq platform. The reads were mapped onto the MGAS315 core genome using the bowtie tool. Samtools mpileup [10] was used for SNP discovery. The resulting VCF file and the GenBank file from the MGAS315 reference genome were used by snp-search to generate the SNP database. snp-search *-output -all_or_filtered_snps* was used to generate an 'alignment’ of the concatenated SNPs from each strain in FASTA format. These SNPs represent positions found in positions conserved in all the genomes, sometimes described as the core genome.

**Figure 3:**
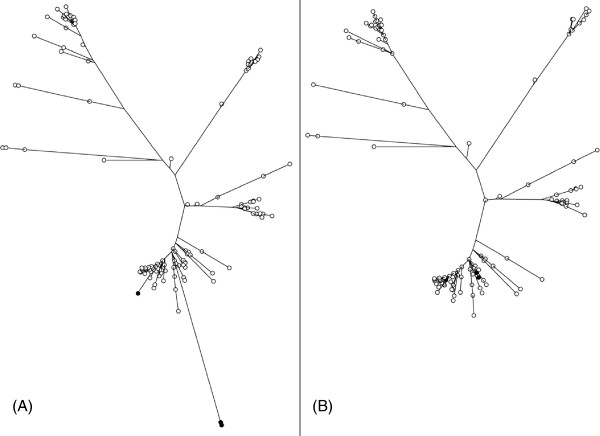
**Maximum-Likelihood (ML) phylogenetic tree of 200 *Streptococcus pyogenes *genomes based on the SNP alignment from the core genome. (A)** Original maximum-likelihood tree **(B)** Tree that shows the shortening of the unusually long branches to the strains represented by black circles. This was made by querying the SNP database generated by snp-search.

To generate a phylogenetic tree, snp-search *-tree* option was used that runs the phylogenetic tree algorithm FastTree 2 to generate a maximum-likelihood (ML) phylogenetic tree of the 200 genomes based on the SNP alignment from the core genome (Figure 3A). One immediate observation from this tree was a long branch composed of a few strains within one of the clades (black circles in Figure 1). This raised questions as to whether certain SNPs were producing unrealistic branch lengths either due to false SNPs or because the SNPs are not caused by neutral evolution. Snp-search was re-run using the *-ignore_snps_on_annotation phage,transposase,transposon,insertion* which queries the SQL database to select only those SNPs not found in genes in mobile elements and likely to undergo recombination. These SNPs were used to remake a multiple alignment file which was then processed by FastTree 2 to produce a ML tree and viewed using Dendroscope [15]. The tree (Figure 3B) shows that the unusually long branches to the strains represented by black circles are shortened so these strains group more consistently with the other strains from the nearest clade. A detailed comparison of the two phylogenies shows that the removal of SNPs in phage-related genes resulted in other more minor changes in the tree.

**Figure 4:**
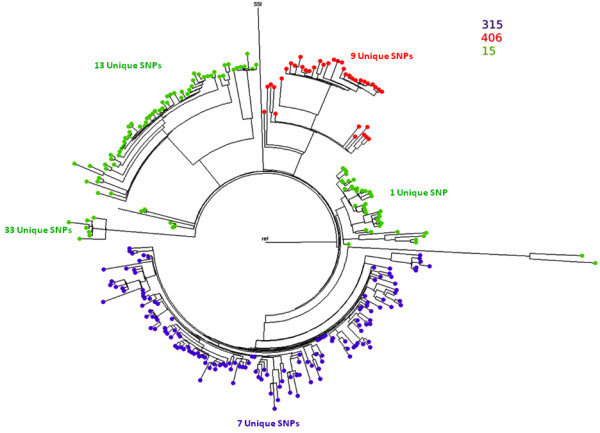
**SNP phylogeny of 295 GAS strains.** snp-search was used to generate the concatenated FASTA file and FastTree was used to generate the tree. Strains are separated according to the number of SNP differences between them. snp-search was also used to output the number of unique SNPs for each distinguishable clade by providing the name of the samples for each clade.

A further 95 GAS strains were sequenced and added to the existing 200 strains. All 295 sequences were mapped to the MGAS315 reference genome, variants called and snp-search was used to generate the tree. MLST data extracted *in* silico showed almost all strains were differentiated into three Sequence Types (STs): ST15, ST315 and ST406 (Figure 4). As shown in Figure 4, ST315 and ST406 strains were confined to one clearly differentiated clonal lineage each, whereas the ST15 strains were present in five differentiated lineages. To evaluate the utility of SNPs for the assessment of genetic diversity between the different STs, we used snp-search to find the SNPs shared only within each clade.

As shown in Figure 4, ST315 clade has 7 unique SNPs, ST406 has 9 and ST15 clades has 1,13 and 33 unique SNPs. The snp-search output for ST315 clade strains is shown in Table 1. For each unique SNP, snp-search identifies whether it is in a coding region or not, and if it is then it reports whether the SNP is synonymous or non-synonymous. Additional information such as the gene and amino acid information is also given.

### Commands

Snp-search is implemented as an easy-to-use command-line tool. The user does not need experience in Unix, a programming language, or SQL (but to manually query the database the user does need to know some SQL). The user can use the example test sets of plasmid samples (suitable for testing purposes as they are small samples) provided in the github repository. (https://github.com/phe-bioinformatics/snpsearch/tree/master/test_data) to test snp-search functionalities. Two files are provided in the example test set directory: plasmid_test_set.vcf and Reference_file_plasmid_test_set.gbk. The following are some commands that can be used when using snp-search (the required fields are in bold):

1. Create the database:

*snp-search ****-create -name_of_database*** plasmid_test_set.sqlite3 ***–reference_file*** Reference_file_plasmid_test_set.gbk ***-vcf_file*** plasmid_test_set.vcf.

2. Now that you have the database plasmid_test_set.sqlite3, you may want to query it. With a simple command it is possible to:

• Generate a concatenated FASTA file

• Generate a phylogenetic tree to view the evolutionary relationships between your samples

• Ignore SNPs that occur in mobile elements

• Ignore SNPs in between nucleotide positions 2000 and 2500

• Ignore SNPs in samples plasmid1, plasmid4 and plasmid8

• Remove non-informative SNPs (SNPs that are found in all samples)

• Only select SNPs with a GQ quality cuttoff of greater than 25

• Only select SNPs with a SNP QUAL cuttoff of greater than 90 (both GQ and QUAL are quality values derived from the VCF file)

• *snp-search ****-output -all_or_filtered_snps -name_of_database ***plasmid_test_set.sqlite3 *-fasta -cuttoff_genotype* 25 *-cuttoff_snp_qual* 90 *-remove_non_informative_snps -ignore_snps_in_range* 2000..2500 *-ignore_strains* plasmid1,plasmid4,plasmid9 *-ignore_snps_on_annotation* phage,transposase,transposon,insertion *-****out*** plasmid_SNPs_concatenated_file.fasta *-tree -fasttree_path* /usr/local/bin/fastTree

• You may then use any phylogenetic tree viewing software to view your tree.

• Further note: In addition to the concatenated FASTA file, SNP positions are provided in two formats: a simple text file and a tsv file.

3 To select SNPs that are shared only between a set of samples, example samples plasmid2, plasmid7, plasmid10, and GQ quality cuttoff of greater than 25 and SNP QUAL cuttoff of greater than 90 you may run the following command (list the sample names on separate in a file called for example 'samples.txt’):

*snp-search ****-output -name_of_database*** plasmid_test_set.sqlite3 ***-unique_snps -strain*** samples.txt *-cuttoff_genotype 25 -cuttoff_snp_qual 90* ***-out*** unique_snps. tx.t

4 If you like to output all SNPs, with information about each SNP (e.g. if it is a synonymous or non-synonymous SNP) and with the quality cuttoff values used in the previous commands, you may run the following command:

*snp-search ****-output -name_of_database*** plasmid_test_set.sqlite3 ***-info****-cuttoff_genotype 25 -cuttoff_snp_qual 90 ****-out*** plasmid_info_for_my_snps. txt.

5 Furthermore, if you like to query the database, you may do so by opening the SQLite3 database generated by snp-search with sqlite3 in your Unix environment. The following is an example of a SQL query that can be performed on the snp-search database to select all snps that are associated with features annotated with a term containing the phrase 'antibiotic’:

SELECT * FROM *snps* INNER JOIN *features_snps* ON *snps.id* = *features_snps.snp_id* INNER JOIN *features* ON *features.id* = *features_snps.feature_id* WHERE *features.id* IN (SELECT *features.id* FROM *features* WHERE *id* IN (SELECT DISTINCT *features.id* FROM *features* INNER JOIN *annotations* ON *annotations.feature_id* = *features.id* WHERE *annotations.value* LIKE '%antibiotic%'));

## Conclusions

SNP data is now commonly generated, at least for smaller genomes, by mapping whole genome sequencing reads to a reference where both known and novel SNPs can be found. To facilitate and understand these findings, we have developed snp-search, a tool that manages SNP data from high throughput haploid sequence datasets and outputs useful information which in turn can be used to test important biological hypotheses. The main advantages of snp-search are:

Parses VCF file and stores SNPs in a database

Designed simple database schema but powerful in answering complex biological queries

Provides detailed information about each SNP

Multiple SNPs filtering steps provided including filtering on the function of genes within which the SNPs are found.

Has no size limit

Only dependent on sqlite3 to build the database.

Sub-populations of SNPs can be extracted from the database to address specific biological questions, such as whether variation in specific genes is linked to phenotypic differences or does the exclusion of SNPs from horizontally transferred elements generate more reliable phylogenetic inferences.

## Availability and requirements

**Project name:** snp-search

**Project home page:**https://github.com/phe-bioinformatics/snp-search.

**Operating system(s):** Unix

**Programming language:** Ruby

**Other requirements:** SQLite, Ruby 1.8.7 or greater and optionally FastTree 2 for phylogenetic tree construction

**License:** GNU General Public License (GPL)

## Competing interests

The authors declare that they have no competing interests.

## Authors’ contributions

AA drafted the manuscript, AU supervised the project and edited the manuscript and AA and AU implemented the snp-search algorithm. All authors read and approved the final manuscript.
